# The easternmost occurrence of *Mammut pacificus* (Proboscidea: Mammutidae), based on a partial skull from eastern Montana, USA

**DOI:** 10.7717/peerj.10030

**Published:** 2020-11-16

**Authors:** Andrew T. McDonald, Amy L. Atwater, Alton C. Dooley Jr, Charlotte J.H. Hohman

**Affiliations:** 1 Western Science Center, Hemet, CA, United States of America; 2 Museum of the Rockies, Montana State University, Bozeman, MT, United States of America; 3 Department of Earth Sciences, Montana State University, Bozeman, MT, United States of America

**Keywords:** *Mammut pacificus*, Mammutidae, Montana, Irvingtonian, Pleistocene

## Abstract

*Mammut pacificus* is a recently described species of mastodon from the Pleistocene of California and Idaho. We report the easternmost occurrence of this taxon based upon the palate with right and left M3 of an adult male from the Irvingtonian of eastern Montana. The undamaged right M3 exhibits the extreme narrowness that characterizes *M*. *pacificus* rather than *M*. *americanum*. The Montana specimen dates to an interglacial interval between pre-Illinoian and Illinoian glaciation, perhaps indicating that *M*. *pacificus* was extirpated in the region due to habitat shifts associated with glacial encroachment.

## Introduction

The recent recognition of the Pacific mastodon (*Mammut pacificus* (Dooley Jr et al., 2019)) as a new species distinct from and contemporaneous with the American mastodon (*M*. *americanum*) revealed an unrealized complexity in North American mammutid evolution during the Pleistocene. Dooley Jr et al. (2019) distinguished *M*. *pacificus* from *M*.  *americanum* by a suite of dental and skeletal features: (1) upper third molars (M3) and lower third molars (m3) much narrower relative to length in *M*. *pacificus*; (2) basal upper tusk diameter for males of a given age is smaller in *M*. *pacificus*; (3) mandibular tusks and alveoli are always absent in *M*. *pacificus*, while variably present in *M*. *americanum*; (4) six fused sacral vertebrae in later ontogenetic stages in *M*. *pacificus*, while *M*. *americanum* exhibits four to six and usually five; and (5) femur with larger midshaft diameter relative to length in *M*. *pacificus*. Dooley Jr et al. (2019) further determined that, while *M*. *americanum* was widespread, occurring from the Eastern Seaboard to the Rocky Mountain region, south into Mexico, and north into Yukon and Alaska, *M*. *pacificus* was geographically restricted to the Irvingtonian–Rancholabrean North American Land Mammal Age (NALMA) of California (58 specimens) and southern Idaho (three specimens).

Here we report an incomplete cranium, MOR 605, from the Irvingtonian of eastern Montana, which exhibits the diagnostically narrow M3 of *Mammut pacificus*, well outside the range of morphological variation observed in *M*. *americanum*. MOR 605 represents an 850-kilometer geographic range extension for *M*. *pacificus* to the east and north, from the vicinity of Pocatello, Idaho to Miles City, Montana. This occurrence indicates that *M*. *pacificus* inhabited the northern Great Plains prior to the Last Glacial Maximum (Dalton et al., 2020), and might have become restricted to California following glaciation of other regions formerly in its range.

## Locality and Geology

Pleistocene fossils have been reported from the Yellowstone River valley for more than a hundred years; fragments of mammoth were discovered near Glendive, Montana (Hay, 1914; Hay, 1924) as early as 1908 (Wilson & Hill, 2000). One of the more significant fossil localities is the Doeden gravel pit north of the Yellowstone River near Miles City, Montana. This site has been studied by archaeologists from the Museum of the Rockies starting in 1976, when the first fossils were collected (Wilson & Hill, 2000). In 1989, the partial skull of a mastodon was recovered (Wilson & Hill, 2000; Wilson & Hill, 2002; Wilson, McDonald & Hill, 2005; Hill, 2006).

The Doeden gravel pit is located within Pleistocene high-terrace deposits above the Yellowstone River (Colton, Luft & Cormier, 1984; Wilson & Hill, 2000). The base of the gravel pit is approximately 64 m above the Yellowstone River where terrace gravels are overlain by Pleistocene fine-grained sediments (Wilson & Hill, 2000). Pit operators at the Doeden gravel site sample approximately six meters deep into the 20 m of maximum thickness of the main terrace, indicating that the fossils were recovered from the upper three meters of gravel (Wilson & Hill, 2000). The majority of the specimens show light to moderate abrasion, suggesting limited transport time and that the fauna may not represent a single local community type (Wilson & Hill, 2000). 

The Doeden local fauna includes two ground sloths (*Megalonyx jeffersonii* and *Paramylodon harlani*), mammoth (*Mammuthus columbi*), mastodon, giant short-faced bear (*Arctodus simus*), multiple horses (*Equus* sp.), camel (*Camelops* sp.), an antilocaprid, a medium–large sized cervid, and a musk ox (*Bootherium* sp.), which are housed in the collections of Museum of the Rockies at Montana State University in Bozeman, Montana (Wilson & Hill, 2000; Wilson & Hill, 2002; Wilson, McDonald & Hill, 2005). The age of the Doeden gravels are estimated to be between approximately 639 ka and 160 ka (Wilson & Hill, 2000; Wilson & Hill, 2002; Lanphere et al., 2002), based on K-Ar, U-series, and radiocarbon dating of regional stratigraphic sequences and geometric features (Hill, 2006). The presence of mastodon in the Doeden gravels may indicate pre-Wisconsinan age and a forested setting along the Yellowstone River (Hill, 2006). The absence of *Bison* in the faunal assemblage indicates the Doeden gravel pit falls within the Irvingtonian NALMA (Wilson & Hill, 2002; Bell et al., 2004; Froese et al., 2017).

## Materials and Methods

Photogrammetry of MOR 605 was carried out at the Museum of the Rockies using a Panasonic Lumix DC-ZS70S 20.3 Megapixel 4K digital camera. The digital 3-D model was created at Western Science Center; the photogrammetric images were processed in AgiSoft PhotoScan to produce a photogrammetric model, which was further refined using Autodesk Meshmixer. The 3-D digital model is available on MorphoSource under the project name “Montana Mammut”, and may be downloaded by request through Museum of the Rockies.

## Results

### Systematic paleontology

**Table:** 

Proboscidea Illiger, 1811
Mammutidae Hay, 1922
*Mammut* Blumenbach, 1799
*Mammut pacificus* Dooley Jr et al., 2019

**Referred specimen:** MOR 605, nearly complete palate with left and right M3.

**Locality:** MOR locality PL-084, Doeden Gravel Pit, Miles City, Custer County, Montana, USA; specimen collected in 1989.

**Horizon:** unconsolidated gravels in terrace deposits above the Yellowstone River, constrained to between approximately 639 ka and 160 ka (Wilson & Hill, 2000; Wilson & Hill, 2002; Lanphere et al., 2002); Chibanian Age, middle Pleistocene Epoch (Cohen et al., 2013); Irvingtonian North American Land Mammal Age (NALMA), based upon the absence of *Bison* in the associated fauna (Wilson & Hill, 2002; Bell et al., 2004; Froese et al., 2017).

### Description

**Figure 1:**
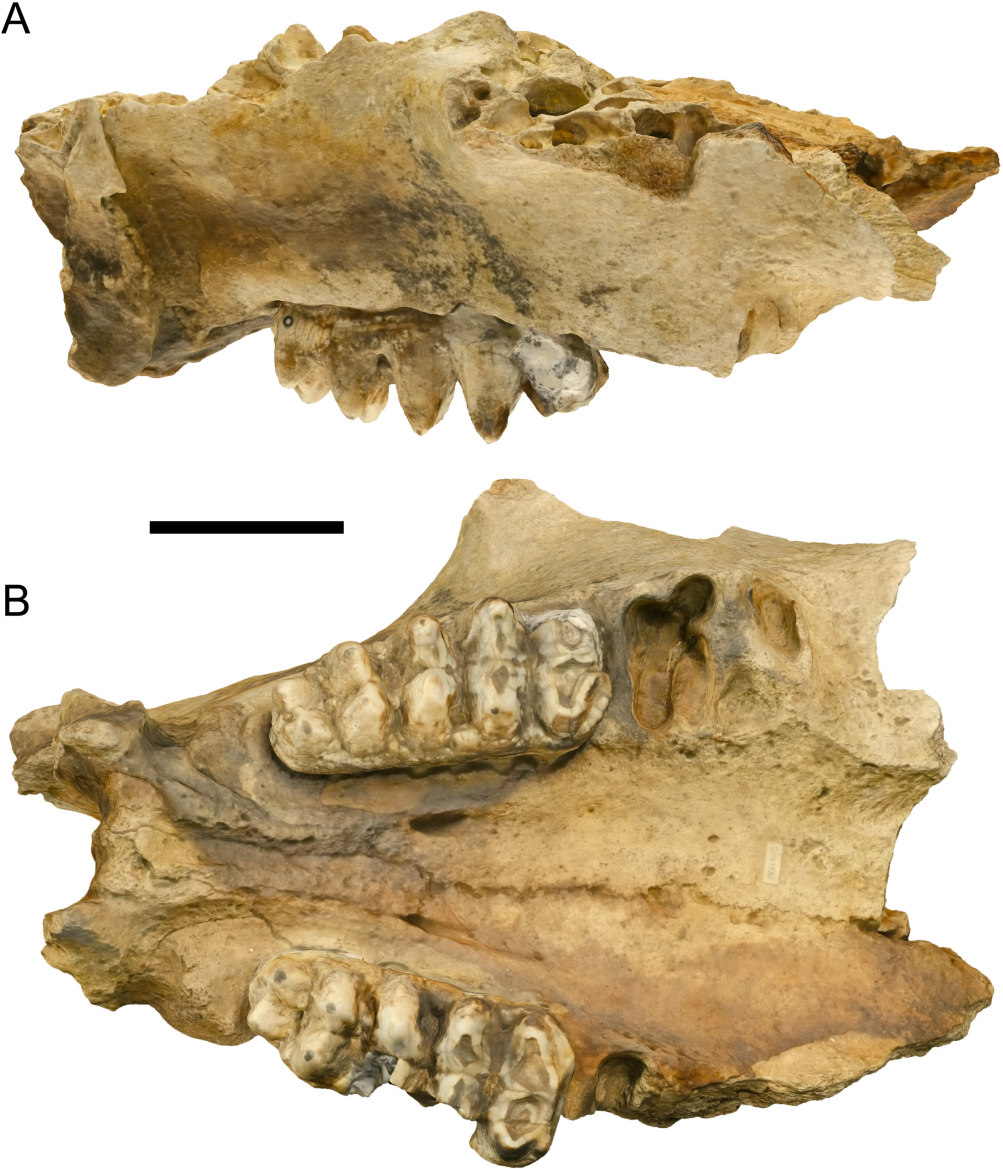
MOR 605, partial cranium of *Mammut pacificus* from Montana. Digital 3-D model in (A) right lateral and (B) ventral orthographic views. Scale bar equals 10 cm.

**Figure 2:**
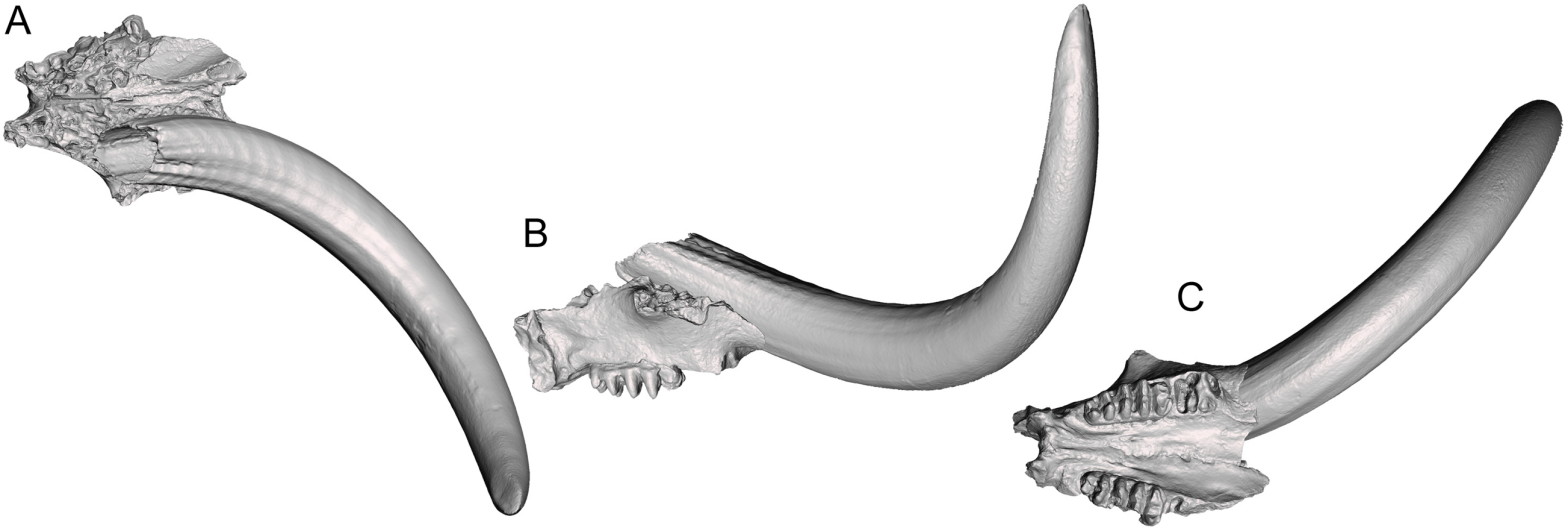
MOR 605 with resized right tusk of WSC 18743. Digital 3-D model in (A) dorsal, (B) right lateral, and (C) ventral orthographic views.

MOR 605 comprises a nearly complete palate with some breakage of the left anterolateral margin and left M3, but with a complete right M3 (Fig. 1). With M2 missing, it is difficult to place the specimen in a Laws Group; however, the wear state of the undamaged right M3 suggests that MOR 605 is probably in Laws Group XIX (32 ± 2 AEY) or XX (34 ± 2 AEY) (Laws, 1966), several years younger than WSC 18743, the adult male holotype of *Mammut pacificus* (LG XXII, 39 ± 2 AEY (Dooley Jr et al., 2019)). To estimate the basal tusk circumference of MOR 605, a digital 3-D model of the complete right tusk of WSC 18743 was resized to fit the partially preserved right alveolus of MOR 605 (Fig. 2). Although this provides at best only a rough estimation of basal tusk circumference, it does suggest that MOR 605 is a male. The approximate basal tusk circumference of MOR 605 is 44 cm, which is in the range reported for adult male *M*. *americanum* (>39 cm) and well outside the range for adult females (<36 cm) (Fisher, 2008; Fisher, 2009; Smith, 2010).

There is a prominent step between the right tusk alveolus and the right maxillary tooth row of MOR 605 (Fig. 1A), similar to WSC 18743 but in contrast to the ontogenetically older adult male *M*. *pacificus* WSC 8817 (LG XXVIII, 55 ± 4 AEY (Dooley Jr et al., 2019)). Distinct steps have been reported in female and juvenile male specimens of *M*. *americanum* (Osborn, 1936; Dooley Jr et al., 2019) hypothesized that the persistence of this feature in adult male *M*. *pacificus* such as WSC 18743 might indicate that maximum tusk size was reached later in ontogeny in *M*. *pacificus* versus *M*. *americanum*. The maxillary tooth rows of MOR 605 are convergent posteriorly (Fig. 1B), in contrast to the parallel tooth rows of WSC 18743 (Dooley Jr et al., 2019). This condition—maxillary tooth rows parallel or convergent posteriorly—is variable in both *M*. *pacificus* and *M*. *americanum* and does not appear to be tied to sex or ontogenetic stage (Osborn, 1936; Dooley Jr et al., 2019).

**Figure 3:**
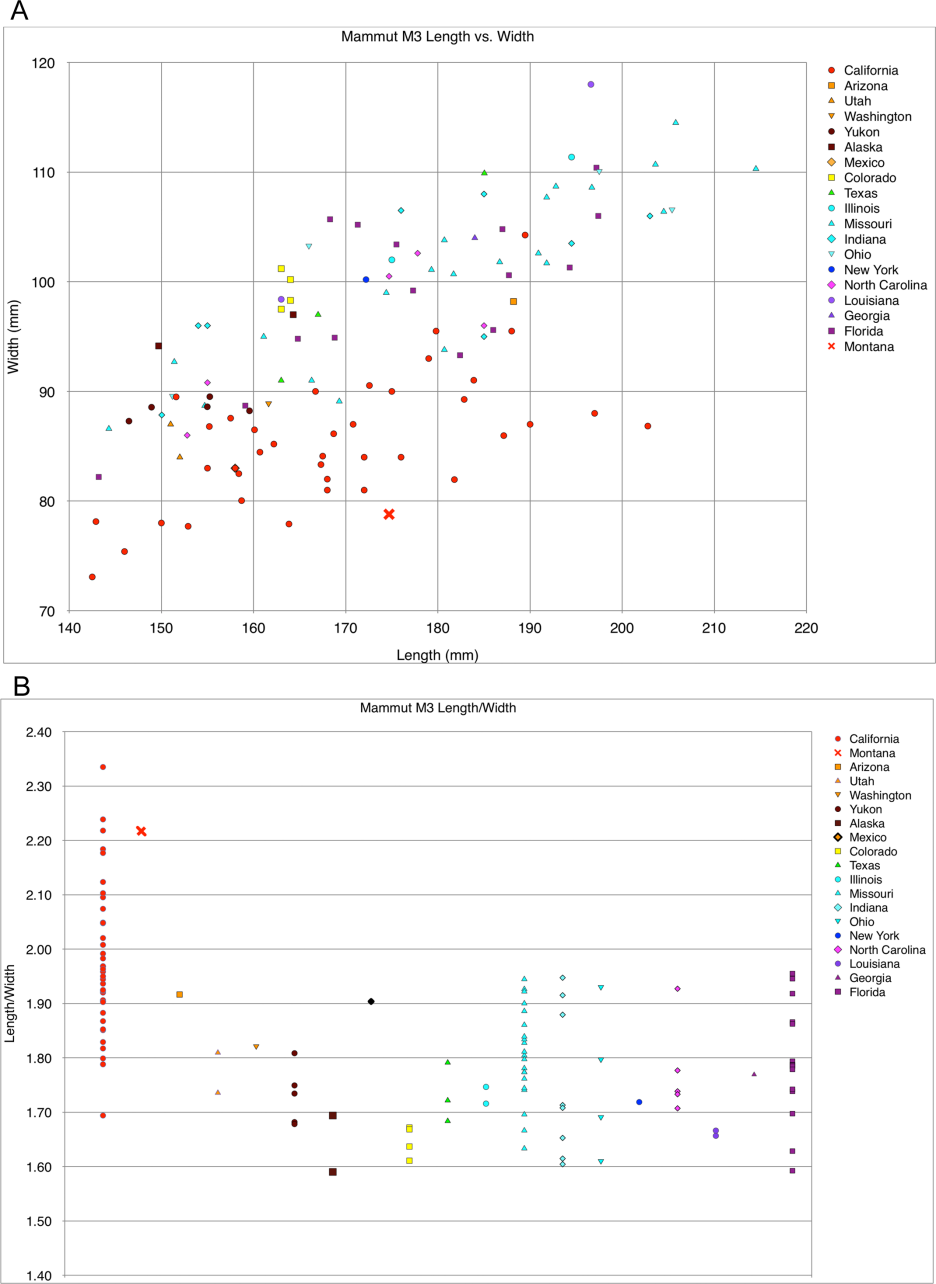
Length and width comparison of *Mammut* M3, by state/province/country. (A) Length versus width of *Mammut* M3; all California specimens (red circles) pertain to *M*. *pacificus*, and MOR 605 (Montana) is marked with a red X. (B) Length/width ratios of *Mammut* M3, with MOR 605 (Montana) again marked with a red X.

The right and left M3 of MOR 605 are pentalophodont, as in a majority of *M*. *pacificus* specimens (24 pentalophodont versus 15 tetralophodont) (Dooley Jr et al., 2019) (Fig. 1). A weakly developed cingulum is present along the anterior and anterolabial margins of the right and left M3, as in all specimens of *M*. *pacificus* and most examples of *M*. *americanum* (Dooley Jr et al., 2019). Both the right and left M3 exhibit smooth enamel, moderate wear on the first loph, lighter wear on the second loph, and almost no wear on the third, fourth, and fifth lophs. Although the left M3 is damaged, measurements can be obtained from the nearly intact right M3 (Table 1): 175 mm total length and 78 mm greatest width across the base of the second loph, with a L:W ratio of 2.24. This is far narrower than any *M*. *americanum* M3 (average L:W ratio = 1.77, maximum = 1.95), and is near the high end of the L:W ratio range of M3 in *M*. *pacificus* (average ratio = 1.98, maximum = 2.33) (Dooley Jr et al., 2019) (Fig. 3). The relatively great narrowness of the right M3 supports the referral of MOR 605 to *M*. *pacificus* rather than *M*. *americanum*. Previously, and prior to the recognition of *M*. *pacificus* as a distinct species (Dooley Jr et al., 2019), the Doeden *Mammut* has been referred to *M*. *americanum* (Wilson & Hill, 2000; Wilson & Hill, 2002; Wilson, McDonald & Hill, 2005; Hill, 2006).

## Discussion

The age of the Doeden local fauna, which includes MOR 605, falls sometime between approximately 639 ka (based on the underlying Lava Creek B Tuff (Wilson & Hill, 2000; Wilson & Hill, 2002; Lanphere et al., 2002) and 160 ka (based on dates derived from calcretes in terrace deposits along the Tongue River, a tributary of the Yellowstone River (Wilson & Hill, 2000; Wilson & Hill, 2002), placing it in the middle Pleistocene (Cohen et al., 2013). According to Bell et al. (2004), the Irvingtonian NALMA began at approximately 1.35 Ma with the first appearance of *Mammuthus* south of 55°N latitude in North America. The end of the Irvingtonian and beginning of the Rancholabrean NALMA is defined as the first occurrence of *Bison* in North America, and is between 195 ka and 135 ka (Froese et al., 2017). In addition to *Mammut pacificus* (MOR 605), the Doeden fauna includes *Mammuthus columbi*, *Bootherium* sp., *Camelops* sp., a cervid, an antilocaprid, *Equus* sp., *Megalonyx jeffersonii*, *Paramylodon harlani*, and *Arctodus simus* (Hill & Schweitzer, 1999; Wilson & Hill, 2000; Wilson & Hill, 2002; Wilson, McDonald & Hill, 2005). As noted by Wilson & Hill (2002) and Wilson, McDonald & Hill (2005), the absence of *Bison* indicates that the Doeden fauna, which is at approximately 46°N latitude, is Irvingtonian in age. Combining the absence of *Bison* with the absolute age constraints suggests that the Doeden fauna is best considered to be late Irvingtonian.

**Table 1:** Measurements of *Mammut pacificus* specimen MOR 605.

**Anatomical Features**	**Measurements (mm)**
Anteroposterior length of palate along midline suture	396
Mediolateral width of palate between left and right M2 alveoli	170
Anteroposterior length of left M3	173
Anteroposterior length of right M3	175
Labiolingual width of right M3 across the bases of each of the five lophs:	
− First loph	76
− Second loph	78
− Third loph	73
− Fourth loph	63
− Fifth loph	46

**Figure 4:**
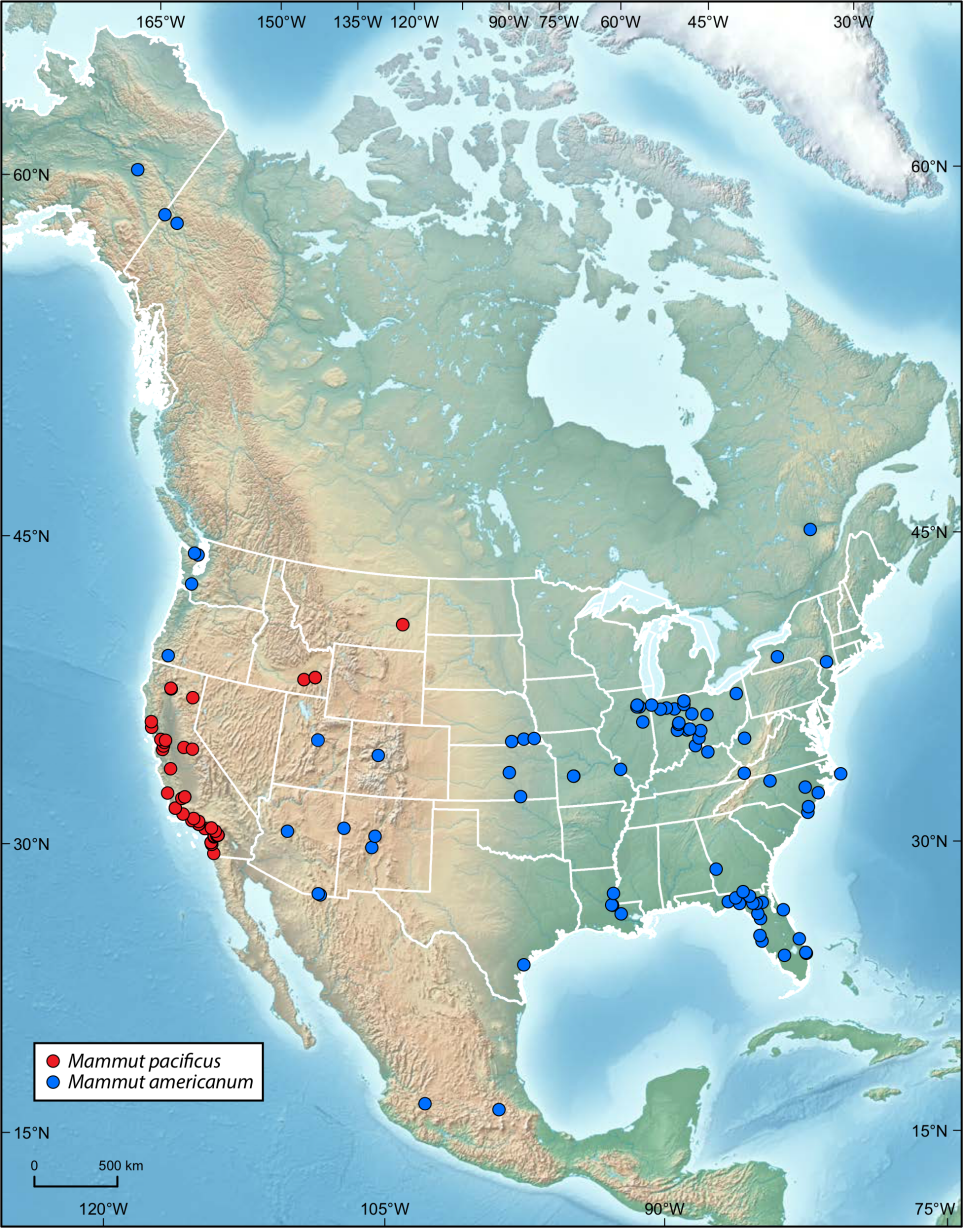
Distribution of specimens of *Mammut pacificus* and *Mammut americanum*. Map of North America showing geographic locations of all sites that have produced specimens of *Mammut pacificus* (red circles), and sites that have produced specimens of *Mammut americanum* used in the charts in Fig. 3 (blue circles). Modified from Dooley Jr et al. (2019) with the addition of *M*. *pacificus* (MOR 605) in Montana.

*Mammut pacificus* is now known from the Irvingtonian of Montana, as well as Irvingtonian sites in Idaho and California (Fig. 4). All known Rancholabrean occurrences of *M*. *pacificus* are in California and Idaho (Dooley Jr et al., 2019). This suggests a contraction of the geographic range of *M*. *pacificus* around the Irvingtonian-Rancholabrean transition, perhaps tied to glaciation in the northern part of its range. Zazula et al. (2014) ascertained a similar scenario for the extinction of *M*. *americanum* in Alaska and Yukon at approximately 75 ka, as habitats changed from boreal woodlands and wetlands to more arid steppe-tundra with expanding glaciation. Although recent studies of specimens from New York, Indiana, Missouri, Florida, and Texas have shown that the diet of *M*. *americanum* was more flexible than previously thought (Green, DeSantis & Smith, 2017; Smith & DeSantis, 2018), American mastodons were nevertheless largely dependent on the availability of browse, including bark and leaves, in forested settings. If Pacific mastodons adhered to the same dietary preferences, then they too would be vulnerable to habitat changes wrought by glaciation.

The eastern Montana plains region was subject to several glacial intervals throughout the Pleistocene. The aforementioned absolute age constraints of the Doeden local fauna at Miles City (∼639 ka–160 ka (Wilson & Hill, 2000; Wilson & Hill, 2002; Lanphere et al., 2002)) place it between two glacial pulses. One of these is a pre-Illinoian glaciation that deposited the upper unit of the Archer till; this glaciation reached as far south as Glendive, only about 100 km to the northeast (Fullerton et al., 2004). This glacial interval is younger than 778 ka, and has a minimum age of 639 ± 2 ka based upon the absolute age of the overlying Lava Creek B Tuff (Lanphere et al., 2002; Fullerton et al., 2004), which also defines the maximum age of the Doeden local fauna. MOR 605 therefore must postdate this glacial interval.

**Figure 5:**
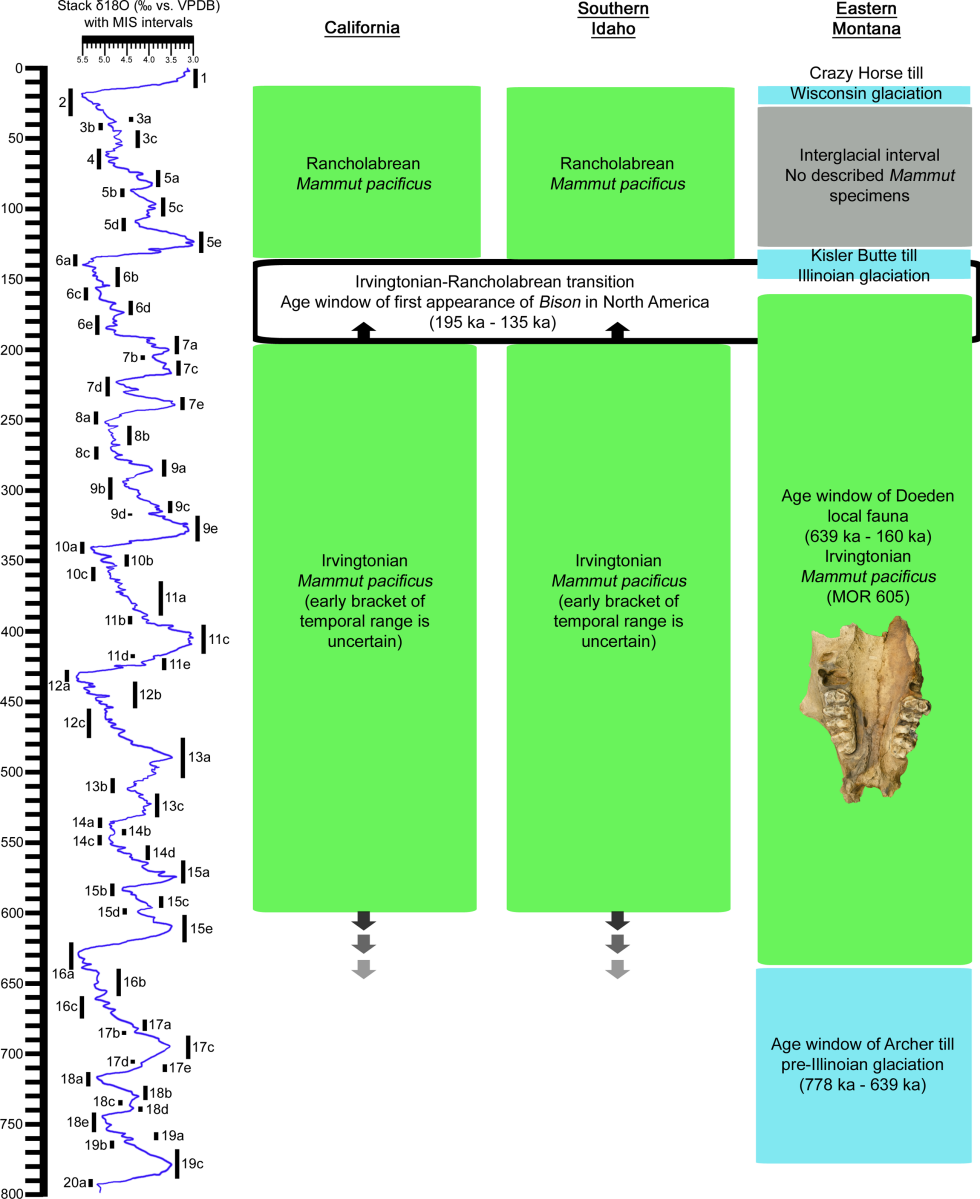
Temporal distribution of *Mammut pacificus* in California, Idaho, and Montana. Ages on the left are in thousands of years. The *δ* ^18^O curve and Marine Isotope Stages (MIS) were drawn after data in Fig. 3 of Railsback et al. (2015). The age data for the first appearance of *Bison* in North America are from Froese et al. (2017). Chronological data for the three geographical areas are from the following sources: California (Pajak, Scott & Bell, 1996; Dooley Jr et al., 2019), Idaho (Gazin, 1935; Izett, Obradovich & Mehnert, 1988; Pinsof, 1991; Dooley Jr et al., 2019), and Montana (Wilson & Hill, 2000; Wilson & Hill, 2002; Lanphere et al., 2002; Fullerton et al., 2004).

The later of the two glacial pulses is an Illinoian glaciation that deposited the Kisler Butte till in eastern Montana around 140 ka (Fullerton et al., 2004), which also stretched as far south as the Glendive area. As noted by Fullerton et al. (2004), there is a gap of approximately 500,000 years between the pre-Illinoian glacial interval that deposited the Archer till and the Illinoian glacial interval that deposited the Kisler Butte till. Based on the age of the Doeden local fauna and mastodon paleoecology in other regions of North America, *Mammut pacificus* inhabited eastern Montana during this interglacial interval and perhaps was extirpated from this part of its geographic range with the advent of Illinoian glaciation (Fig. 5).

This scenario would explain the presence of Pacific mastodon in Montana during the Irvingtonian and apparent restriction of its geographic range to California and Idaho in the Rancholabrean. However, numerous additional questions arise. Did *Mammut pacificus* reestablish itself in Montana during subsequent interglacial intervals, or did *M*. *americanum* supplant it? Did the two species’ ranges ever overlap, and if so, did they interbreed? How did the ranges of the two species fluctuate in response to environmental shifts throughout the Pleistocene? Apart from specimens of *M*. *pacificus* from Idaho, to our knowledge the described mastodon specimens geographically closest to MOR 605 are specimens of *M*. *americanum* from northern Utah (Miller, 1987) and northern Colorado (McDonald et al., 2010; Fisher et al., 2014). *M*. *americanum* remains from the Wasatch Plateau in Utah (Miller, 1987) and Snowmass in Colorado (Fisher et al., 2014; Mahan et al., 2014; Sertich et al., 2014) are Rancholabrean in age and postdate MOR 605. However, a specimen from Ken-Caryl in Colorado might be between 200,000 and 130,000 years old, which would make it possibly late Irvingtonian in age (McDonald et al., 2010).

McDonald et al. (2010) mentioned but did not describe a small number of mastodon specimens from Montana, Idaho, Utah, Wyoming, Colorado, Arizona, and New Mexico, while Dooley Jr et al. (2019) further noted the limited number of specimens from Washington, Oregon, and Nevada. A fuller understanding of the paleobiogeographic histories of *M*. *pacificus* and *M*. *americanum* must await additional and more precisely dated specimens from the Great Plains, Rocky Mountain region, and Pacific Northwest, as well as more precise dates for *M*. *pacificus* specimens from California. In conjunction with the description of specimens such as MOR 605, micro/mesowear and stable isotope studies of *M*. *pacificus* teeth will further elucidate mastodon paleoecology during the Pleistocene.

## Conclusions

MOR 605, the palate and M3s of an adult male mastodon, represents the first occurrence of *Mammut pacificus* in Montana and the easternmost record of the species. *M*. *pacificus* might have suffered a local extirpation in Montana due to the onset of Illinoian glaciation, and subsequently became restricted to California and Idaho. This and other paleobiogeographic questions remain difficult to answer without additional specimens from regions of North America, such as Montana, where mastodon fossils are rare and further paleoecological investigations.

## Institutional abbreviations

MOR: Museum of the Rockies, Bozeman, Montana, USA
WSC: Western Science Center, Hemet, California, USA.

## Additional Information and Declarations

### Data Availability

The following information was supplied regarding data availability: The mastodon fossil described is accessioned at the Museum of the Rockies in Bozeman, Montana, USA: specimen number MOR 605. The digital 3D model of MOR 605 is available at MorphoSource (licensed under CC BY NC). https://morphosource.org/Detail/ProjectDetail/Show/project_id/1025. - MOR 605 (palate): https://doi.org/10.17602/M2/M115603. - composite of MOR 605 (palate) and downscaled WSC 18743 (tusk): https://doi.org/10.17602/M2/M115604. - WSC 18743 (tusk): https://doi.org/10.17602/M2/M68673.
